# *Escherichia coli* phage ΦPNJ-9 adheres to mucus via a variant Hoc protein

**DOI:** 10.1128/jvi.01789-24

**Published:** 2024-12-26

**Authors:** Kailai Fu, Jiaqi Cui, Yao Li, Yuhan Zhang, Yang Wang, Jiaoling Wu, Xinru Chen, Feng Xue, Jianluan Ren, Jianjun Dai, Fang Tang

**Affiliations:** 1 Key Laboratory of Animal Bacteriology, Ministry of Agriculture, College of Veterinary Medicine, Nanjing Agricultural University 261674 , Nanjing, Jiangsu, China; 2 School of Life Science and Technology, China Pharmaceutical University 56651 , Nanjing, Jiangsu, China; Michigan State University, East Lansing, Michigan, USA

**Keywords:** phage, *Escherichia coli*, Hoc protein, mucus, adhesion, binding site

## Abstract

Phages, as antagonists of bacteria, hold significant promise for combating drug-resistant bacterial infections. Their host specificity allows phages to target pathogenic bacteria without disrupting the gut microbiota, offering distinct advantages in the prevention and control of intestinal pathogens. The interaction between the phage and the gut plays a crucial role in the efficacy of phage-mediated bacterial killing. However, the mechanisms underlying these interactions remain poorly understood. In this study, we demonstrate that the clinically isolated T4-like phage, ΦPNJ-9, effectively adheres to the intestinal mucosa *in vivo*. This adhesion is mediated by the phage’s Hoc protein, which interacts with MUC2 in the mucus. The Hoc protein of ΦPNJ-9 represents a variant, consisting of only three domains and lacking Domain 3, in contrast to phage T4. The key interacting sites on ΦPNJ-9 Hoc are amino acids S183, L184, and T185 within Domain 2. Displaying Domain 2 of ΦPNJ-9 Hoc on the surface of M13 phage significantly enhances its adhesion to the intestinal mucosa. Additionally, we identify fucose residues in MUC2 as the critical binding sites for the phage. Through this adhesion, the phage occupies the intestinal niche, thereby protecting the mucosal layer from pathogenic *Escherichia coli* infections. Our findings highlight the role of Hoc proteins in phage adhesion to intestinal mucus and the variation in binding sites, providing key insights for phage-based strategies aimed at preventing and controlling intestinal pathogens.

**IMPORTANCE:**

The rise in antibiotic-resistant pathogenic bacteria has sparked renewed interest in phage therapy as a promising alternative, particularly for targeting intestinal pathogens due to phage’s host specificity. However, clinical applications have revealed that many phages are ineffective in eliminating bacteria within the gut, primarily due to the complex interactions between the phage and the gut environment. However, the mechanisms underlying these interactions remain poorly understood. Our previous study demonstrated that a T4-like phage adheres to the intestinal mucosa through the interaction between its Hoc protein and MUC2 in the mucus. Whether this model is widespread among T4-like phages remains unknown. Here, we characterize a variant Hoc protein from a T4-like phage, and identify new binding sites within this protein. Our findings suggest that the interaction between Hoc and MUC2 is likely common, but the critical binding sites vary depending on the specific phage.

## INTRODUCTION

As antibiotic resistance continues to rise, bacteriophage (phage) has garnered increasing attention as a potential treatment for bacterial infections. The emergence of superbugs under natural selection, coupled with the lengthy development timelines for new, highly effective antibiotics, underscores the need for alternative therapies (1, 2). Phage, specific virus that infects and inhibits host bacteria, can be easily sourced from nature, offering a promising solution to mitigate the risks of antibiotic resistance (3–5). Studies have demonstrated that phages targeted against pathogenic bacteria can effectively reduce bacterial load and alleviate disease symptoms, including intestinal inflammation (6, 7). For example, a single day of oral treatment with a three-phage cocktail in LF82-colonized mice significantly reduced the number of *Adherent-invasive Escherichia coli* (AIEC) in feces and in the adherent flora of intestinal sections. Additionally, a single dose of the cocktail alleviated dextran sodium sulfate-induced colitis symptoms in conventional mice colonized with the LF82 over a 2-week period (8). In another study, a single oral dose of phage X1, administered 6 h post-infection, eliminated *Yersinia enterocolitica* (*Y. enterocolitica*) in 33.3% of mice (15 out of 45), and dramatically reduced the bacterial load from approximately 10^7^ CFU/g to 10^3^ CFU/g after 18 h compared to untreated mice (9). Furthermore, in fish models, administration of IME-JL8 (1 × 10^7^ PFU) effectively protected carp infected with a double median lethal dose (2 × 10^9^ CFU/carp) and reduced pro-inflammatory cytokine levels in carp infected with a lethal dose (10).

Global virome data suggest that more than 90% of human enteric viruses are phage (11–13). Phage plays a crucial role in maintaining gut homeostasis by regulating gut microbiota and achieving ecological balance through host lysis or modulating bacterial metabolism and activity (14–16). In the intestine, the ratio of phage to bacteria is considered to be about 1:1, with the highest ratio in the mucus layer, which may be about 4.4 times higher than in the surrounding environment, suggesting that phage accumulates in the intestinal mucus layer (17). Mucus serves as the first line of defense in the intestine. Goblet cells secrete mucus to separate bacteria from epithelial cells, promoting bacterial clearance and reducing intestinal inflammation and enteric infections (18–20). The main components of mucus are mucins, highly glycosylated macromolecular proteins classified into secreted (MUC2, MUC5AC, MUC5B, MUC6, MUC19, MUC7) and membrane-bound (MUC1, MUC3A, MUC3B, MUC4, MUC12, MUC13, MUC16, MUC17, and MUC20) categories based on structural properties. MUC2, synthesized by goblet cells, is the most critical component of intestinal mucus (21, 22). MUC2 is a highly glycosylated protein, with its backbone binding various O-type oligosaccharide side chains. These O-glycosylations, which include GalNAc, Gal, GlcNAc, NeuAc, and Fuc residues, protect mucins from proteolysis and play a key role in host-microbiota interactions (23–25). The Bacteriophage Adherence to Mucus (BAM) model, proposed by Barr (17, 26), suggests that Hoc protein on the capsid surface of T4 phage binds to the mucus layer, increasing the likelihood of phage contact with bacterial hosts, thus reducing pathogen colonization and protecting intestinal epithelial cells from bacterial infection *in vitro*. Building on this, our previous study showed that the T4-like phage ΦPNJ-6, isolated from chicken feces, also displays a Hoc protein on its capsid. This protein shares 87.8% homology with T4 phage Hoc in terms of amino acid sequence and facilitates phage adherence to intestinal mucus by binding to MUC2. This interaction enables the phage to occupy a mucosal niche and reduce *Enterotoxigenic E. coli* (ETEC) colonization *in vivo*. The key binding sites are E29 and G33 in Domain 1 of the Hoc protein and the fucose residues of MUC2 (27).

The Hoc protein of T4 phage is located at the center of each hexameric capsid and contains four domains: three Ig-like domains and a non-Ig-like domain at the C-terminus. The non-Ig-like domain is not believed to be involved in T4 phage adhesion to mucus (17, 28). Hoc appears to be broadly present across the T4-like phage family. Similar Hoc-like fibrous molecules have also been identified in the capsids of T5 and other phages (29). However, structural differences among these phages are significant, with some exhibiting domain deletions. In this study, we isolated an *E. coli* phage, ΦPNJ-9, from poultry farm sewage. Whole-genome sequencing revealed that ΦPNJ-9 is a T4-like phage, containing a *hoc* gene. The nucleotide sequence identity of the *hoc* gene in ΦPNJ-9 was 65.07% and 70.29% compared to T4 and ΦPNJ-6, respectively. Unlike phage T4 and ΦPNJ-6, which contain four domains in their Hoc proteins, the Hoc protein of ΦPNJ-9 lacks the third domain. We investigated the adhesion and protective ability of ΦPNJ-9 to intestinal mucosa *in vivo*, specifically against pathogenic *E. coli* infection. Additionally, we identified a novel key binding site in the Hoc protein of ΦPNJ-9 that interacts with MUC2.

## RESULTS

### Whole-genome sequencing and analysis of phage ΦPNJ-9

**Fig 1:**
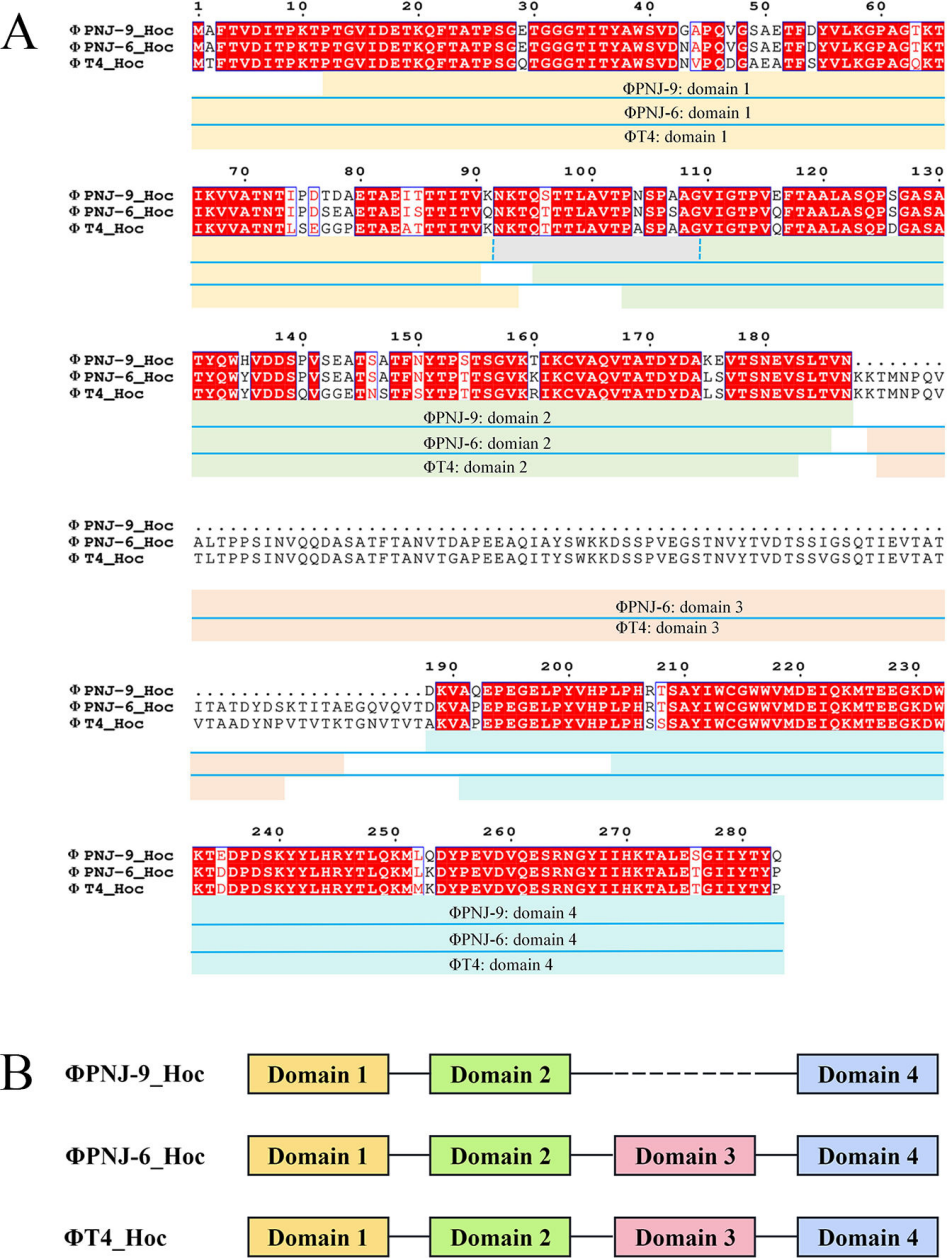
Hoc protein of different phages. (**A**) Amino acid sequence alignment of Hoc protein among phages ΦPNJ-9, ΦPNJ-6, and T4. This figure is created by clustalw and ESPript. (**B**) Hoc domains of ΦPNJ-9, ΦPNJ-6, and T4. The image was created by Figdraw.

The genome of ΦPNJ-9 is 170,071 bp (GenBank accession number: PQ100635, containing 284 open reading frames (ORFs). ΦPNJ-9 is classified as a T4-like phage, exhibiting the highest homology to *E. coli* phage ΦPNJ-6, sharing 99.75% nucleotide sequence identity with 98% coverage. No toxin, resistance, or lysogeny-related genes were identified in the genome of ΦPNJ-9, suggesting that this is a virulent phage suitable for therapeutic applications. The genome of ΦPNJ-9 contains a *hoc* gene, which shares 65.07% and 70.29% nucleotide sequence identity with the *hoc* genes of phage T4 and ΦPNJ-6, respectively (Fig. 1A). The Hoc protein of phage T4 contains four complete domains (three Ig-like domains: Domain 1, Domain 2, Domain 3 and one non-Ig-like domain: Domain 4) (28), whereas the Hoc protein of ΦPNJ-9 consists of only three domains, lacking Domain 3 (Fig. 1B).

### Hoc protein and mucus mediate adhesion of phage ΦPNJ-9 to mouse intestinal mucosa

**Fig 2:**
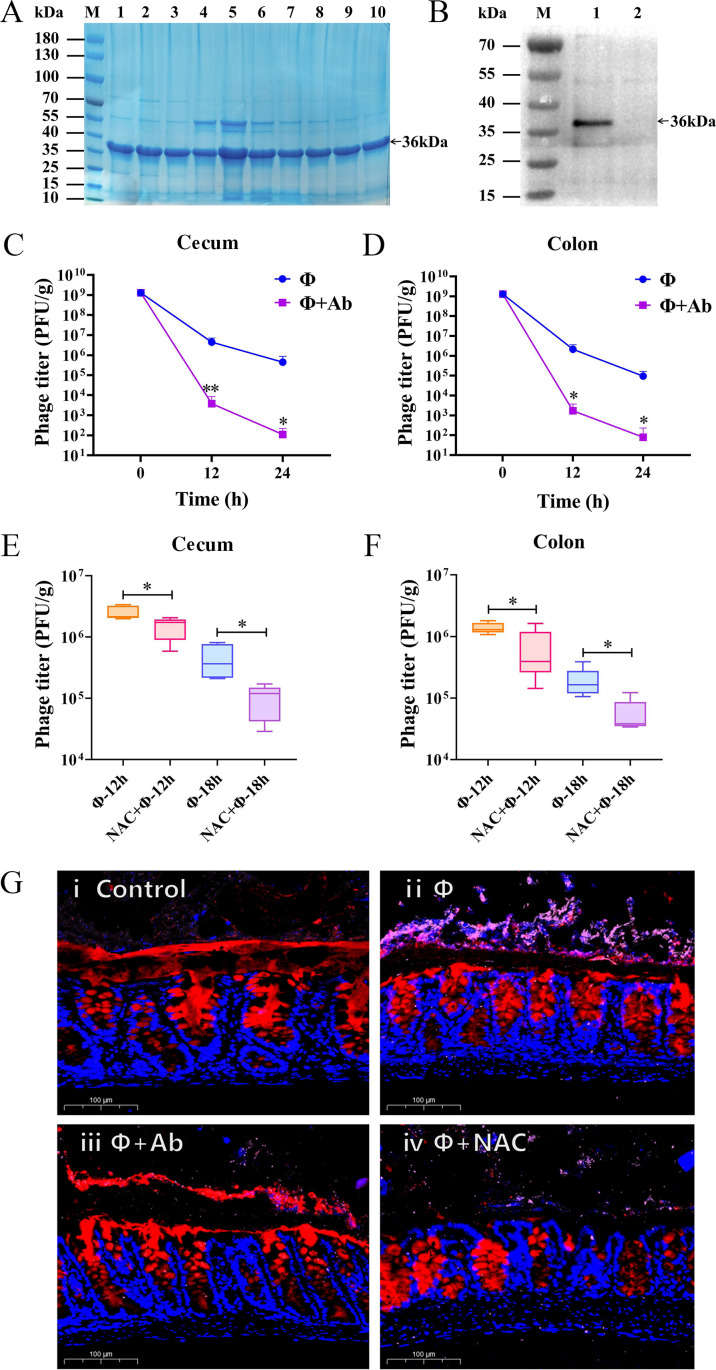
Role of Hoc protein and mucus in phage adhesion to the intestinal mucosa. (**A**) SDS-PAGE analysis of purified ΦPNJ-9 Hoc protein. Lanes 1–10 represent purified Hoc protein samples. (**B**) Western blot assay to test the specificity of murine polyclonal Hoc antibody. Lane 1, ΦPNJ-9; Lane 2, ΦPNJ-6. (**C and D**) Phage titers in mouse cecum (**C**) and colon (**D**) following treatment with free phage or Hoc antibody-blocked ΦPNJ-9. *, *P* < 0.05; **, *P* < 0.01. (**E and F**) Phage titer in the mouse cecum (**E**) and colon (**F**) treated with or without NAC. *, *P* < 0.05. (**G**) Immunofluorescence images of mouse colon sections. Red: MUC2, blue DAPI (cell nucleus), and pink: phage ΦPNJ-9. Scale bar: 100 µm. (ⅰ) Normal mouse colon without any treatment; (ⅱ) Phage adhesion to mouse colon; (ⅲ) Phage adhesion blocked by Hoc antibody, resulting in a significant decrease in phages adherence; and (ⅳ) NAC treatment leading to mucus removal, resulting in a significant reduction in phage adherence.

To determine whether the Hoc protein mediates the adhesion of ΦPNJ-9 to the intestinal mucosa, we tested the adhesion of phages blocked by hoc antibodies in mice. Recombinant Hoc protein of ΦPNJ-9 was successfully expressed and purified (Fig. 2A). Polyclonal antibodies were generated by immunizing mice with the purified Hoc protein and Western blot analysis confirmed that the antibodies exhibited strong immunogenicity against the Hoc protein (Fig. 2B). Subsequently, we administered the phage, either blocked with Hoc antibodies or free to mice by intragastric administration. Mice were dissected at 12 and 24 h post-administration to determine the phage titers in the cecum and colon. The results demonstrated that in both cecum and colon, phage titers in the antibody-blocked group were reduced by more than three orders of magnitude (*P* < 0.05) at both time points compared to the free phage group (Fig. 2C and D). These findings suggest that the Hoc protein plays a critical role in mediating the adhesion of ΦPNJ-9 to the intestinal mucosa.

To investigate whether phage adhesion to mouse intestinal mucosa is associated with mucus, we examined the ability of phage ΦPNJ-9 to adhere to mucus-depleted mucosa. Mice were treated with N-acetyl-l-cysteine (NAC), a mucolytic agent, to remove mucus from their intestines. Phage ΦPNJ-9 was then administered intragastrically to both NAC-treated and untreated mice. Mice were dissected 12 and 18 h post-administration to determine phage titers in the cecum and colon. The results showed that the phage titers in the cecum of untreated mice were 2.55 × 10^6^ and 4.67 × 10^5^ PFU/g at 12 and 18 h, respectively, while the phage titers in the cecum of NAC-treated mice were significantly decreased at both time points (*P* < 0.05), with values of 1.48 × 10^6^ and 1.01 × 10^5^ PFU/g, respectively (Fig. 2E). Similarly, in the colon, after 12 and 18 h, the phage titers in untreated mice were 1.39 × 10^6^ and 1.91 × 10^5^ PFU/g, respectively. In contrast, the phage titers in the NAC-treated mice were significantly reduced at both time points (*P* < 0.05), with values of 6.59 × 10^5^ and 5.62 × 10^4^ PFU/g, respectively (Fig. 2F). These findings suggest that the adhesion of ΦPNJ-9 to the intestinal mucosa of mice is closely associated with mucus.

To further validate the interaction between Hoc protein and mucus, we performed an indirect immunofluorescence assay *in vivo*. Mice were divided into four groups: (i) control group, intragastrically administered with phosphate-buffered saline (PBS); (ii) phage group, intragastrically administered phage ΦPNJ-9; (ii) phage block group, intragastrically administered phage ΦPNJ-9 blocked with Hoc antibody; and (iv) NAC-treated group, first treated with NAC followed by intragastric administration of phage ΦPNJ-9. Fluorescent imaging revealed that the control group was covered with a substantial amount of mucus (Fig. 2G-ⅰ). After phage administration, a large number of phage adhered to the surface of the intestinal mucosa (Fig. 2G-ⅱ). In the phage-blocked group, phage adherence to the intestinal mucosa was dramatically reduced, and the phage was scarcely observed on the mucosal surface (Fig. 2G-ⅲ). In the NAC-treated group, where mucus was nearly removed from the mucosal surface, minimal phage adhesion was observed (Fig. 2G-ⅳ). These results further confirm that both Hoc protein and mucus play a crucial role in phage adhesion to the intestinal mucosa.

### Hoc protein of ΦPNJ-9 can bind to MUC2

**Fig 3:**
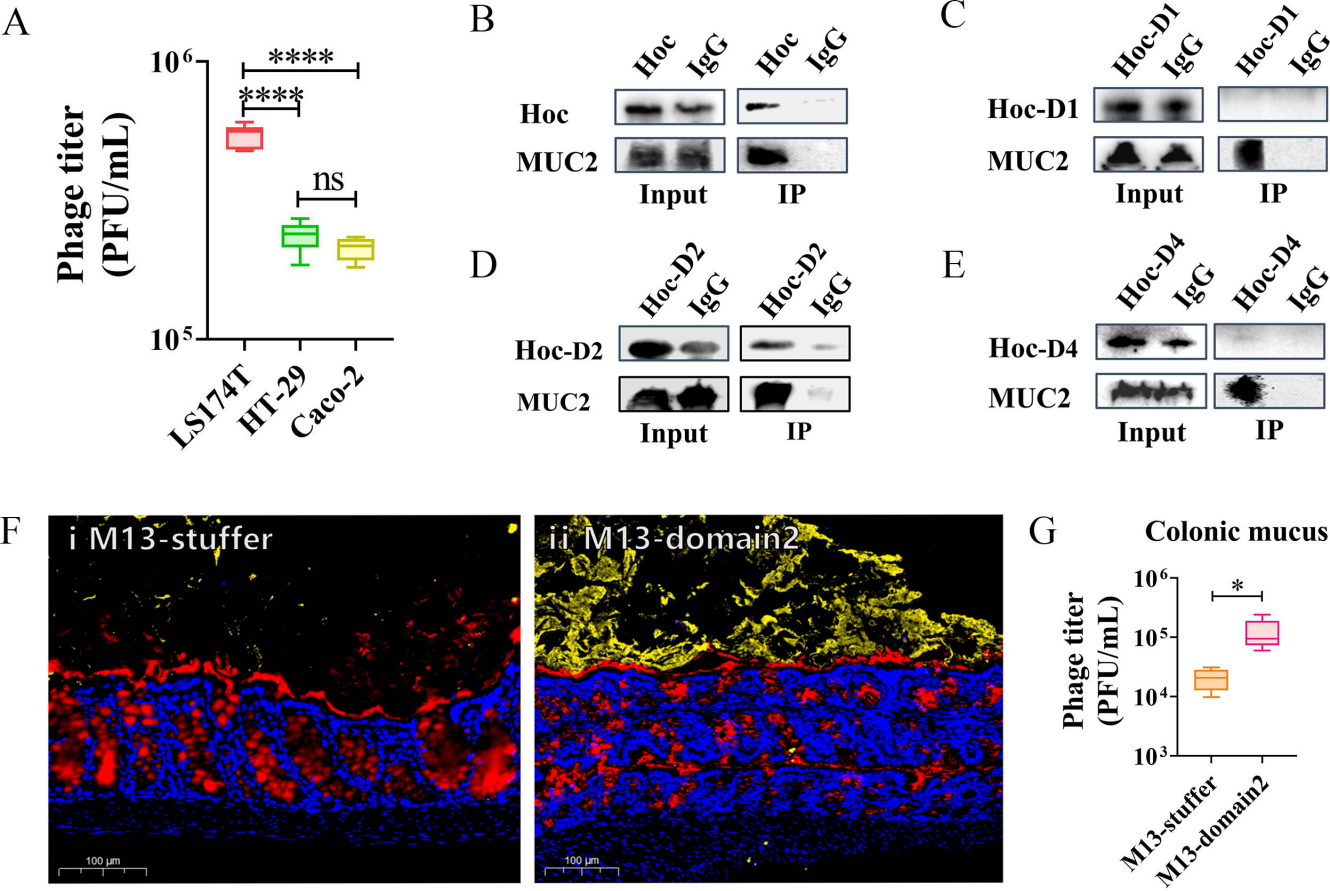
Hoc protein Domain 2 of ΦPNJ-9 binds to MUC2. (**A**) Adhesion of ΦPNJ-9 on three colon cancer cells. ****, *P* < 0.0001. Co-IP assays showing interaction between MUC2 and Hoc protein (B), Domain 1 (**C**), Domain 2 (**D**), and Domain 4 (**E**). (**F**) Immunofluorescence images of mouse colon sections. Red: MUC2; blue: DAPI (cell nuclei); and yellow: recombinant M13 phage. Scale bar: 100 µm. (ⅰ) M13 phage displaying stuffer sequence showed minimal adhesion to the mouse colon. (ⅱ) M13 phage displaying Hoc protein Domain 2 exhibited significant adhesion to the mouse colon. (**G**) Quantification of recombinant M13 phage adhesion to the mouse colon. *, *P* < 0.05.

The major component of intestinal mucus is MUC2 (21, 22, 30), and our previous study demonstrated that Hoc protein of ΦPNJ-6 can bind to MUC2 (27). Therefore, we investigated whether the Hoc protein of ΦPNJ-9 could also interact with MUC2. It has been documented that three intestinal cells LS174T, HT-29, and Caco-2 secrete varying levels of MUC2, with LS174T cells exhibiting significantly higher MUC2 mRNA expression than HT-29 and Caco-2 cells (31). We assessed the adhesion ability of ΦPNJ-9 to these cell lines and found that ΦPNJ-9 exhibited the strongest adhesion to LS174T cells (Fig. 3A), indicating that phage adhesion to cells may be linked to MUC2 expression.

We then collected MUC2 secreted by LS174T cells and performed co-immunoprecipitation (Co-IP) experiments to assess the interaction between Hoc protein and MUC2. The results demonstrated that Hoc binds to MUC2 (Fig. 3B). To further identify the critical domains of the Hoc protein involved in MUC2 binding, we expressed and purified three distinct domains of the Hoc protein from ΦPNJ-9: Domain 1 (P12-G109), Domain 2 (N92-N187), and a non-Ig-like Domain 4 (D188-Q283). Co-IP assays with MUC2 revealed that Domain 1 did not bind to MUC2 (Fig. 3C), while Domain 2 successfully interacted with MUC2 (Fig. 3D). Domain 4, the non-Ig-like domain, also failed to bind to MUC2 (Fig. 3E). These findings indicate that Domain 2 of the Hoc protein is the critical region responsible for binding to MUC2.

To further validate the interaction between MUC2 and Hoc Domain 2, we utilized phage display technology. M13 phages, which display functional components on their capsid proteins, were used for this purpose (32). We displayed the nucleic acid sequence of Domain 2 of the Hoc protein from ΦPNJ-9, as well as an unrelated sequence (a Stuffer sequence inserted between *Sfi* I and *Not* I restriction sites in the MCS region of the pCANTAB 5E plasmid vector), on the PIII protein of M13 phage (Table S1). Mice were then intragastrically administered to either M13 phages displaying Domain 2 or the unrelated sequence. After 7 h, mice were dissected, and the amount of phage adhering to their colons was observed. The results showed that M13 phage displaying the unrelated sequence scarcely adhered to the mouse colon (Fig. 3F-ⅰ), while M13 phage displaying Domain 2 adhered extensively to the colon (Fig. 3F-ⅱ). Moreover, the titer of M13 phages in mouse colonic mucus was measured, revealing that M13 phage displaying Domain 2 had significantly higher titer in the colonic mucus compared to M13 phages displaying unrelated sequences (*P* < 0.05) (Fig. 3G), These results confirm that Domain 2 of the Hoc protein plays a key role in phage adhesion to the intestinal mucosa.

### Phage and Hoc protein do not affect MUC2 expression

**Fig 4:**
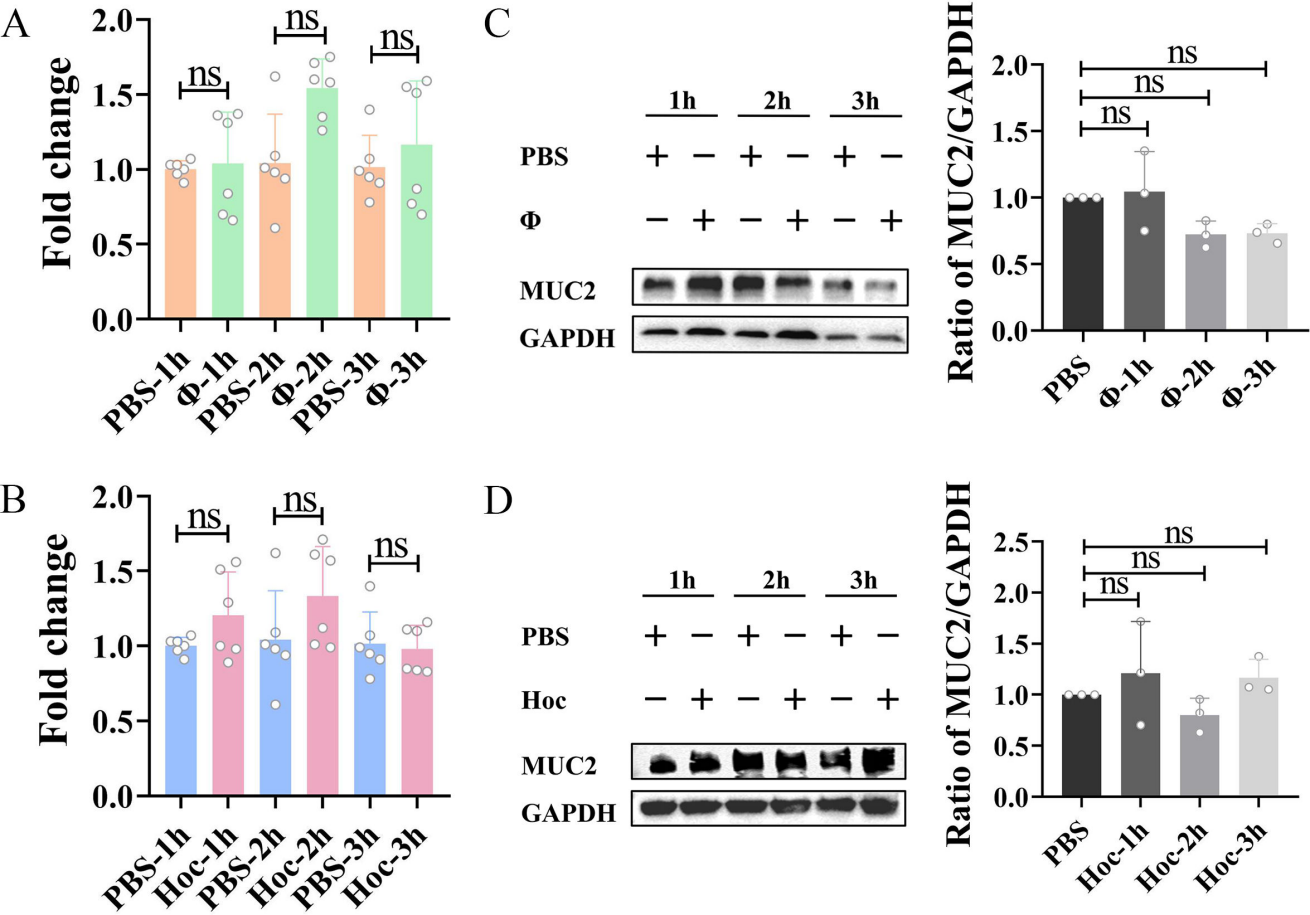
Efffect of phage and Hoc protein on MUC2 expression in LS174T cells. mRNA transcription levels of *Muc2* in LS174T cells after incubation with ΦPNJ-9 (**A**) or Hoc protein (**B**). Expression levels of MUC2 in LS174T cells after incubation with ΦPNJ-9 (**C**) or Hoc protein (**D**). ns, no significant difference.

Our previous study showed that phage ΦPNJ-6 could upregulate MUC2 expression (27). In this study, we aimed to investigate whether ΦPNJ-9 and its Hoc protein have similar effects on MUC2 expression. We incubated phage ΦPNJ-9 or purified Hoc protein with LS174T cells for 1, 2, or 3 h. The mRNA transcription levels of *Muc2* in LS174T cells were measured by real-time RT-PCR, and the total protein was collected for Western blot analysis to assess MUC2 expression. The results showed that neither ΦPNJ-9 nor the purified Hoc protein affected the mRNA transcription levels of *Muc2* (Fig. 4A and B) nor the protein expression (Fig. 4C and D), indicating that the expression of MUC2 is not regulated by ΦPNJ-9 or its Hoc protein.

### S183, L184, and T185 of Hoc protein and fucose residues of MUC2 are the key binding sites

**Fig 5:**
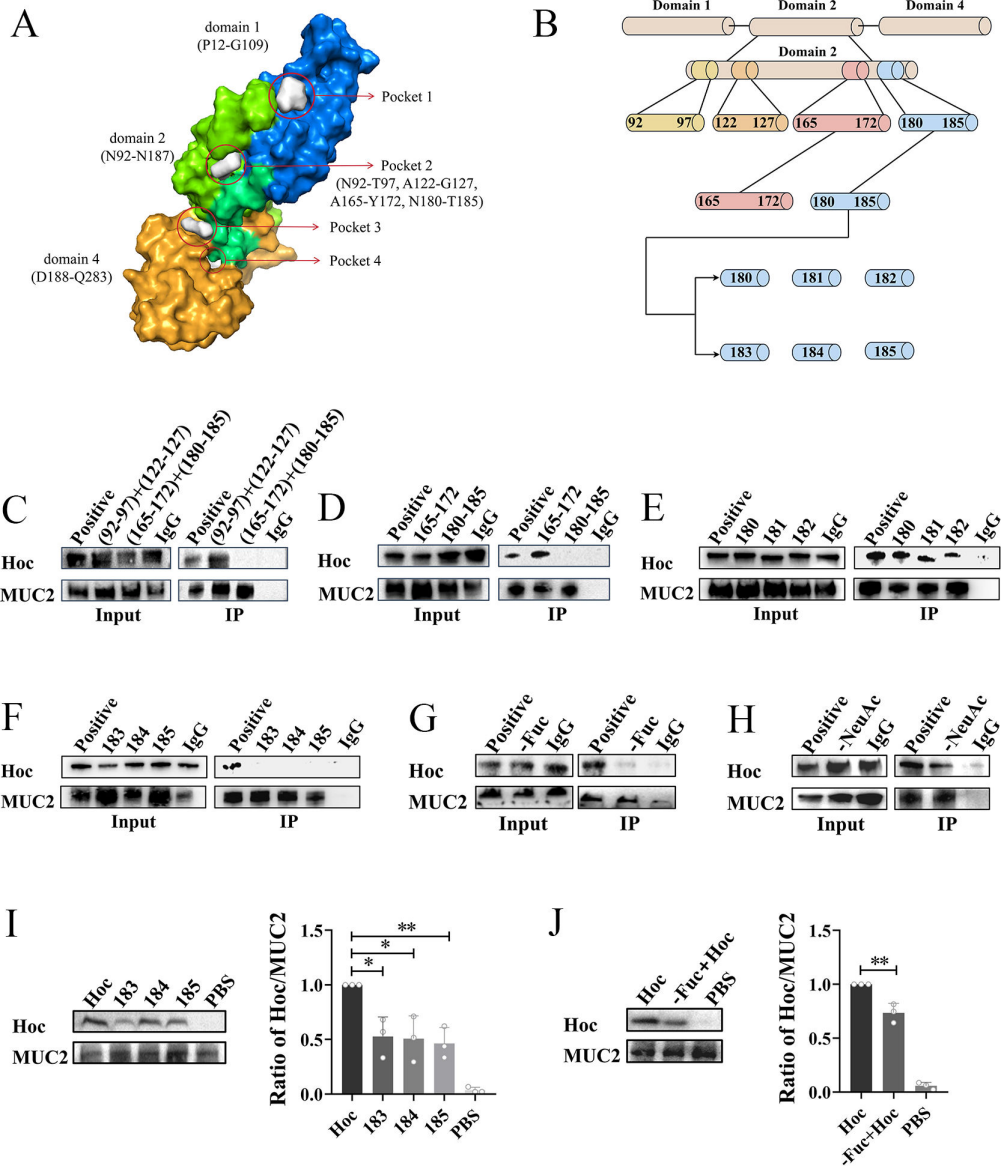
Critical binding sites mediating the interaction between the Hoc protein and MUC2. (**A**) The structure of Hoc protein: contains three domains and a pocket is completely in Domain 2. (**B**) Schematic representation of amino acid site mutations in the Hoc protein. Image created using Figdraw. (**C**) Co-IP between MUC2 and (N92-T97) + (A122-G127) or (A165-Y172) + (N180-T185) mutant Hoc proteins. (**D**) Co-IP between MUC2 and (A165-Y172) or (N180-T185) mutant Hoc proteins. (**E**) Co-IP between MUC2 and N180, E181, or V182 mutant Hoc proteins. (**F**) Co-IP between MUC2 and S183, L184, or T185 mutant Hoc proteins. (**G**) Co-IP between α1–2,4,6 fucosidase O-treated MUC2 and Hoc protein. (**H**) Co-IP between α2–3,6,8,9 neuraminidase A-treated MUC2 and Hoc protein. (**I**) Binding of S183, L184, or T185 mutant Hoc proteins to LS174T cells. *, *P* < 0.05; **, *P* < 0.01. (**J**) Binding of Hoc protein to LS174T cells treated with α1–2,4,6 fucosidase O. **, *P* < 0.01.

To further identify critical binding sites of the Hoc protein that interact with MUC2, we constructed a model of the Hoc protein using I-TASSER and predicted the binding pocket using POCASA based on this model (33, 34). The results showed a binding pocket in the Domain 2 of Hoc (Fig. 5A). We then mutated 26 critical amino acids within this pocket, specifically (N92-T97), (A122-G127), (A165-Y172), and (N180-T185) (Fig. 5B). First, two mutant Hoc proteins, (N92-T97) + (A122-G127) and (A165-Y172) + (N180-T185), were constructed and subjected to Co-IP assays with MUC2. The results showed that the (A165-Y172) + (N180-T185) mutant protein could not bind to MUC2, while the (N92-T97) + (A122-G127) mutant protein was able to bind (Fig. 5C), indicating that the critical binding site lies within (A165-Y172) + (N180-T185). We then constructed additional mutant Hoc proteins for (A165-Y172) and (N180-T185), and performed Co-IP assays with MUC2. The results indicated that the (N180-T185) mutant protein failed to bind to MUC2, while the (A165-Y172) mutant protein retained binding capability (Fig. 5D), further confirming that the critical binding site is within N180-T185. Finally, we constructed six individual mutant Hoc proteins (N180, E181, V182, S183, L184, and T185). Co-IP assays with MUC2 revealed that N180, E181, and V182 mutant proteins were able to bind to MUC2 (Fig. 5E), while S183, L184, and T185 mutant proteins could not (Fig. 5F). These findings indicate that the key binding sites on Hoc are S183, L184, and T185.

To identify the critical binding sites of MUC2 for the Hoc protein, we focused on the glycosylation pattern of MUC2, which includes abundant sialic acid (NeuAc) and fucose (Fuc) residues at its termini (25, 35). We selectively removed the fucose and sialic acid residues from MUC2 using α1–2,4,6 fucosidase O and α2–3,6,8,9 neuraminidase A, respectively, followed by a Co-IP assay with Hoc. The results demonstrated that MUC2 lacking the fucose residue failed to bind Hoc (Fig. 5G), while MUC2 lacking the sialic acid residue could bind to Hoc (Fig. 5H), suggesting that the fucose residue on MUC2 is essential for Hoc binding.

To further confirm the critical binding sites for Hoc on MUC2, we assessed the adhesion capacity of various Hoc proteins to LS174T cells. We incubated LS174T cells with either wild-type Hoc protein or Hoc proteins mutated at S183, L184, or T185. After 1.5 h of incubation, the Hoc protein adhered to the cells was detected by Western blot assay. The results showed robust adhesion of wild-type Hoc to LS174T cells, whereas the S183, L184, or T185 mutants exhibited significantly reduced binding (*P* < 0.05) (Fig. 5I). Additionally, Hoc protein was incubated with either α1–2,4,6 fucosidase O-treated or untreated LS174T cells, and cell-bound Hoc was detected by Western blot. The binding of Hoc was significantly reduced in fucosidase-treated cells compared to untreated cells (*P* < 0.01) (Fig. 5J). These results confirm that S183, L184, and T185 on Hoc, as well as fucose residues on MUC2, are essential for this interaction.

### Hoc enhances the bactericidal effect of phage ΦPNJ-9 *in vivo*

**Fig 6:**
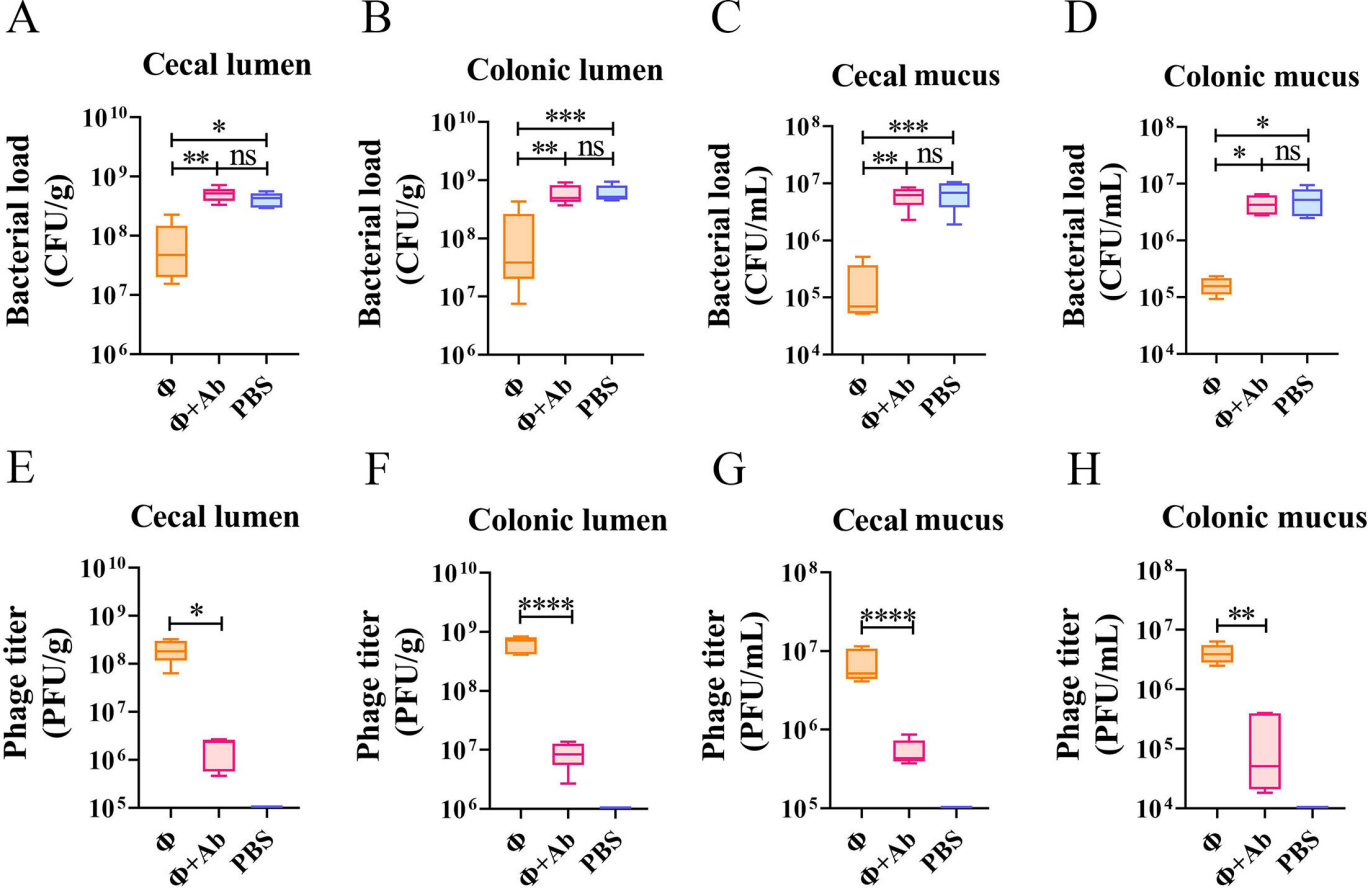
Bacterial and phage loads in mice intestines. Bacterial loads in the cecal lumen (**A**), colonic lumen (**B**), cecal mucus (**C**). and colonic mucus (**D**) in mice; phage loads in the cecal lumen (**E**), colonic lumen (**F**), cecal mucus (**G**), and colonic mucus (**H**) in mice. *, *P* < 0.05; **, *P* < 0.01; ***, *P* < 0.001; and ****, *P* < 0.0001.

To assess the role of Hoc protein in modulating the bactericidal efficacy of phages in the intestine, we performed preventive experiments using a murine model. Mice were intragastrically administered either ΦPNJ-9, ΦPNJ-9 pre-blocked with Hoc antibody, or PBS. After 10 h, the mice were challenged with ETEC SH232, and subsequently dissected 7 h post-infection to quantify the bacterial and phage loads in the cecal and colonic lumen and mucus layer. The results revealed no significant difference in bacterial load between the PBS and antibody-blocked phage group in both the cecal and colonic lumen and mucus layer. However, the phage-treated group exhibited significantly lower (*P* < 0.01) bacterial loads in cecal lumen (7.61 × 10^7^ CFU/g) and colonic lumen (1.21 × 10^8^ CFU/g) compared to the antibody-blocked phage group (cecal lumen: 5.07 × 10^8^ CFU/g; colonic lumen: 5.97 × 10^8^ CFU/g) (Fig. 6A and B). Similarly, the bacterial loads in the cecal mucus layer (1.81 × 10^5^ CFU/mL) and colonic mucus layer (1.62 × 10^5^ CFU/mL) of the phage-treated group was significantly lower (*P* < 0.05) compared to the antibody-blocked phage group (cecal mucus layer: 6.08 × 10^6^ CFU/mL; colonic mucus layer: 4.47 × 10^6^ CFU/mL) (Fig. 6C and D). Phage loads were significantly higher (*P* < 0.05) in the cecal and colonic lumen of the phage-treated group (caecal lumen: 2.05 × 10^8^ PFU/g; colonic lumen: 6.30 × 10^8^ PFU/g) compared to the antibody-blocked group (cecal lumen: 1.75 × 10^6^ PFU/g; colonic lumen: 8.97 × 10^6^ PFU/g) (Fig. 6E and F). In the mucus layers, phage loads in the phage-treated group (cecal mucus layer: 7.07 × 10^6^ PFU/mL; colonic mucus layer: 4.13 × 10^6^ PFU/mL) were also significantly higher (*P* < 0.01) than those in the antibody-blocked group (cecal mucus layer: 5.36 × 10^5^ PFU/mL; colonic mucus layer: 1.74 × 10^5^ PFU/mL) (Fig. 6G and H). These results indicate that Hoc protein enhances phage adhesion to the intestinal mucosa, enabling better ecological niche occupation and significantly improving the bactericidal effect against pathogenic *E. coli*, thereby offering protection to the intestinal mucosa.

## DISCUSSION

Our results demonstrate that the capsid protein Hoc of ΦPNJ-9 binds to MUC2 in the intestinal mucus layer. This interaction facilitates phage adhesion within the mucus, thereby mitigating bacterial invasion of the intestinal mucosa. Chin et al. employed surface plasmon resonance to quantitatively assess the binding affinity between glycans and surface-immobilized Hoc proteins of T4 phage, revealing that Hoc of T4 exhibits strong binding properties to fucose (34). Similarly, Wu et al. demonstrated that fucose residues as critical binding sites for the interaction between Hoc of phage ΦPNJ-6 and MUC2 (27). Our findings align with these studies, suggesting that fucose serves as preferred binding sites for Hoc proteins of T4-like phages.

Compared to the Hoc protein of phage T4, the Hoc protein of ΦPNJ-9 lacks Domain 3. Our findings identify Domain 2 as the key interaction site, while our previous study highlighted Domain 1 as the critical binding site for Hoc protein of ΦPNJ-6 (27). Additionally, Chin et al. identified a D246N mutation in the third Ig-like domain of T4 Hoc, which removes an acidic residue (aspartic acid) and replaces it with a neutral residue (asparagine), altering the binding affinity to mucin glycans (36). Our previous work demonstrated that amino acids E29 and G33 in Domain 1 of ΦPNJ-6 Hoc are critical for MUC2 binding (27). In contrast, we identified amino acids S183, L184 and T185 in Domain 2 of ΦPNJ-9 Hoc as the primary interaction sites, which diverge from previous findings. While the amino acid residues at positions 29, 33, and 183–185 are conserved in the Hoc proteins of ΦPNJ6 and ΦPNJ9, the differences in domain architecture—four domains in ΦPNJ6 Hoc versus three in ΦPNJ9 Hoc—likely influence protein folding, residue spatial arrangement, and the shape and positioning of the binding pocket, thereby impacting interactions with target proteins. Structural predictions suggest that in ΦPNJ6 Hoc, the binding pocket conformation may favor residues E29 and G33 for interaction, while in ΦPNJ9 Hoc, the conformation of the binding pocket likely positions residues S183, L184, and T185 as the primary interaction site. Our previous research has shown that Hoc proteins are widely distributed among phages targeting various Enterobacteriaceae bacteria, with considerable variability in their structure and sequence (27). This variability suggests that while Hoc proteins universally interact with MUC2, the key binding sites may differ significantly due to structural and sequence differences. Furthermore, our prior study showed that Hoc protein of ΦPNJ-6 upregulates MUC2 mRNA and protein expression levels (27). However, neither ΦPNJ-9 nor its Hoc protein affected mRNA and protein expression levels, indicating that the effect of individual phages and their Hoc proteins on eukaryotic cells are highly specific, despite certain similarities. The underlying mechanism remains to be elucidated. This also underscores the need to consider the distinct impacts of individual phages on the host organism in phage therapy.

Upon examing the loading of antibody-blocked and unblocked phages in the mouse intestine, we observed significant differences dependent on the presence of bacteria. In the absence of bacteria, phage loading in the cecum and colon differed by more than three orders of magnitude between the blocked and unblocked groups (27). However, in the presence of bacteria, the difference in the phage loading between the blocked and unblocked groups in both the cecum and colon was about one to two orders of magnitude. This discrepancy is likely due to the replication of phage in the presence of bacteria, where newly replicated phages are not blocked by Hoc antibodies, thereby diminishing the difference in phage loading between the two groups. Furthermore, we observed that, in the presence of bacteria, phage ΦPNJ-9 levels in the colonic mucus layer were significantly lower in the blocked group compared to the unblocked group, differing by more than one order of magnitude. Interestingly, in our previous study, no significant difference in the colonic mucus layer phage load was observed between blocked and unblocked groups for phage ΦPNJ-6 (27). We hypothesize that this discrepancy may stem from differences in the replication rates of two phages. To test this, we determined the one-step growth curves for both phages. The results revealed burst sizes of ΦPNJ-6 are approximately 2.4 times higher than that of ΦPNJ-9 (Fig. S1), indicating that ΦPNJ-6 may proliferate more rapidly in the intestine due to its higher replication rate. As a result, when reaching the colon, a larger number of ΦPNJ-6 phages that were not blocked by antibodies likely accounted for the smaller differences observed compared to the unblocked group.

In this study, we confirmed that the interaction between Hoc and MUC2 played a dominant role in phage adhesion to the intestinal mucosa. However, blocking Hoc or removing MUC2 did not completely abolish the phage’s ability to adhere, suggesting that additional factors may contribute to phage colonization in the intestine. Green et al. reported a novel phage whose lytic cycle is enhanced in intestinal environments. The tail fiber protein of this phage binds human heparan sulfated proteoglycans, localizing the phage to the epithelial cell surface and positioning it near its bacterial host, thus functioning as a locational targeting mechanism (37). Lehti et al. demonstrated the binding and penetration of *E. coli* PK1A2 phage into live eukaryotic neuroblastoma cells *in vitro*, where the phage interacts with cell surface polysialic acid, a structure similar to its bacterial receptor (38). These studies suggest that different structural proteins on the phage may interact with various glycoproteins in the intestine. Beyond these direct interactions, the physiological structure of the gut can also influence phage colonization and bactericidal efficacy, such as intestinal spatial heterogeneity affecting phage-bacteria coexistence (39).

In conclusion, we isolated a virulent phage ΦPNJ-9, which binds to fucose residues on MUC2 in the mucus layer of the intestinal mucosa through key amino acids S183, L184, and T185 on the Hoc protein. This binding enables the phage to adhere to the intestinal mucosa, resist invasion by pathogenic *E. coli*, and provide protective effect on the intestine. Our findings confirm the universality of the Hoc-MUC2 interaction and highlight the diversity of Hoc protein action sites. These insights are valuable for advancing the study of phage interactions with the intestine and for the development of phage therapy against intestinal pathogens.

## MATERIALS AND METHODS

### Strains, phages, and plasmids

ETEC SH232 was isolated from the feces of Chinese piglets with diarrhea. Phage ΦPNJ-9 was isolated from poultry farm sewage. DH5α competent cells (TSC-C01, China) and BL21 (DE3) competent cells (TSC-E01, China) were purchased from Nanjing Qingke Biotechnology Co., Ltd. The expression vector used for prokaryotic protein expression was pET-28a. *E. coli* TG1 strain, M13KO7 helper phage, and pCANTAB 5E plasmid vector were gifts from Professor Huiying Ren of Qingdao Agricultural University.

### Whole-genome sequencing of phage ΦPNJ-9 and structural analysis of Hoc protein

Genomic DNA of ΦPNJ-9 was extracted using a phage genomic DNA extraction kit (ABigen, China). First-generation sequencing was performed on an ABI 3730 (Applied Biosystems, USA) at Tsingke Biotech, Beijing, China. For additional details on genomic sequencing of ΦPNJ-9 and structural analysis of Hoc protein, see Text S1.

### Hoc antibody blocking assay *in vivo*

Four-week-old BALB/c mice were administrated phage ΦPNJ-9, either with or without Hoc antibody blocking. After 12 and 24 h, cecal and colonic tissues were collected, and phage loads were quantified. For further details on prokaryotic expression and purification of Hoc protein, preparation of polyclonal Hoc antibodies, and Hoc antibody blocking assay *in vivo*, see Text S1.

### Affinity test of phage to mucus *in vivo*

Four-week-old BALB/c mice were randomly divided into two groups and treated with or without NAC prior to ΦPNJ-9 administration. After 12 and 18 h post-phage gavage, cecal and colonic tissues were collected, and phage loads were quantified. For further details on the affinity test of phage to mucus *in vivo*, see Text S1.

### Adhesion of phage to different intestinal cells

LS174T, HT-29, and Caco-2 cells were cultured in six-well plates. Phage ΦPNJ-9 was added and incubated for 2 h. After incubation, the cells were washed and scraped, and the phage titer was determined. For further details on the adhesion of phage to different intestinal cells, see Text S1.

### Adhesion of Hoc protein and its mutant to cells

LS174T cells were cultured in six-well plates until a monolayer was formed. If necessary, α1–2,4,6 fucosidase O was added and incubated to remove fucose residues from MUC2. Wild type, or mutant Hoc protein, or PBS, was then added to the cells and incubated for 1.5 h. After incubation, cell samples were collected, and the affinity of proteins to cells was assessed by Westen blot. For further details on the construction and expression of mutant Hoc protein, removal of fucose residues from MUC2, and adhesion of Hoc protein and its mutant to cells, see Text S1.

### Co-IP assay

To assess the interaction between wild type Hoc, mutant Hoc, or Hoc domains and MUC2, Co-IP was performed. MUC2 antibody was first bound to A/G Magnetic Beads, followed by the addition of LS174T cell lysate for incubation. After incubation, wild-type Hoc, mutant Hoc, or Hoc domains were added to the mixture. The presence of different Hoc proteins and MUC2 in the samples was then detected by Western blot. For further details on Co-IP assay, see Text S1.

### Immunofluorescent staining *in vivo*

To investigate the adhesion of ΦPNJ-9 or recombinant M13 phage in mouse intestine, mice were divided into different groups. Colonic tissues were then collected, processed into paraffin sections, and subjected to fluorescence staining. For further details on group assignment, M13 phage display, preparation of paraffin sections, and fluorescence staining, see Text S1.

### Effect of phage and Hoc protein on MUC2 expression

LS174T cells were cultured in six-well plates and a monolayer was formed. Hoc protein or phage solution was then added, and the cells were incubated at 37℃ for 1, 2, or 3 h. After incubation, total cellular RNA and protein were extracted. *Muc2* transcription levels by qPCR and MUC2 protein expression were analyzed by Western blot. For further details on the detection of MUC2 transcription and protein expression levels, see Text S1.

### Prevention experiment in mice

Mice were randomly divided into three groups: (i) ΦPNJ-9 + ETEC, (ii) Hoc antibody-blocked ΦPNJ-9 + ETEC, and (iii) PBS + ETEC. After treatment, intestinal mucus and contents were collected, and phage loads were quantified. For further details on the prevention experiments in mice, see Text S1.

### Statistical analysis

Statistical analysis was performed using GraphPad Prism 8.0 software (GraphPad Software, La Jolla, CA, USA). Error bars represent 95% confidence intervals, with the midline indicating the mean ± standard deviation for histograms and line graphs. Data were analyzed using a *t* test or one-way analysis of variance. A *P* value of less than 0.05 was considered statistically significant, as indicated by asterisks (*, *P* < 0.05; **, *P* < 0.01; ***, *P* < 0.001; and ****, *P* < 0.0001).

## Data Availability

The genome sequence of phage ΦPNJ-9 has been deposited in NCBI GenBank under accession code PQ100635. Sequences for Soc, Hoc domains, Hoc, and Hoc mutants are provided in Table S1. Source data are available with this paper.

## SUPPLEMENTAL MATERIAL

Text S1 jvi.01789-24-s0001.docx Supplemental methods.

Figure S1 jvi.01789-24-s0002.tif One-step growth curve.

Supplemental legend jvi.01789-24-s0003.docx Legend for Fig. S1.

Table S1 jvi.01789-24-s0004.xlsx Nucleotide and amino acid sequences in this study.

Table S2 jvi.01789-24-s0005.xlsx Primers in this study.

ASM does not own the copyrights to Supplemental Material that may be linked to, or accessed through, an article. The authors have granted ASM a non-exclusive, world-wide license to publish the Supplemental Material files. Please contact the corresponding author directly for reuse.
