# Supplementary material for: Escherichia coli phage ΦPNJ-9 adheres to mucus via a variant Hoc protein
Source: J Virol. 2024 Dec 26;99(2):e01789-24. doi: 10.1128/jvi.01789-24 (PMC11853027; doi:10.1128/jvi.01789-24)
Supplement: Text S1 — Supplemental methods. [file jvi.01789-24-s0001.docx]

## Geneome sequencing and analysis of phage ΦPNJ-9

DNA libraries were constructed using the Illumina NovaSeq 6000 sequencing platform and the NEBNext Ultra DNA Library Preparation Kit (NEB, USA) for Illumina kits. Whole-genome sequencing analysis was performed by Tianjin Novogene Bioinformation Technology Co., Ltd.

A sequence alignment of the *hoc* genes from ΦPNJ-9, ΦPNJ-6 and T4 phage was conducted using BLAST against the National Center for Biotechnology Information (NCBI) database. Open reading frames (ORFs) of the phage were predicted using NCBI ORFfinder (https://www.ncbi.nlm.nih.gov/orffinder/). Phage Lysogeny was assessed using Prophage Hunter (<https://pro-hunter.>genomics.cn). Integrative conjugative elements (ICEs) and integrative mobile elements (IMEs) were predicted using ICEfinder (https://bioinfo-mml.sjtu.edu.cn/ICEfinder/). Virulence factors and antibiotic resistance genes were predicted using VirulenceFinder-2.0 and Resfinder (https://www.genomicepidemiology.org/).

## Structural analysis of Hoc protein

The Hoc structure model of ΦPNJ-9 was predicted using the I-TASSER online tool (<https://zhanggroup.org/>I-TASSER/). The model with the highest C-core and TM-core greater than 0.5 was selected to ensure accurate topology. Protein binding pockets were then predicted using the POCASA tool (http://altair.sci.hokudai.ac.jp/g6/service/pocasa/). Based on the selected structural model, the predicted structure and binding pockets were further analyzed using PyMOL.

## Prokaryotic expression and purification of Hoc protein and its domains

primers were designed using the primer design tool from Nanjing Novozymes Biotechnology Co., Ltd (https://crm.vazyme.com/cetool/singlefragment.html), and all primers are listed in Table S2. The target genes were amplified by PCR using ΦPNJ-9 as a template. The pET-28a plasmid vector was digested with *Bam*H I (1010S, Takara, Japan) and *Xho* I (1094S, Takara, Japan) at 37℃ for 15 min. The PCR-amplified target gene was ligated into the double-digested plasmid via homologous recombination. The recombinant plasmid was then transformed into DH5α competent cells and cultured at 180 rpm at 37℃ for 1.5 h. Bacteria were plated on LB-Kana plates and incubated overnight at 37℃. Positive clones were selected and cultured in liquid LB medium. The target fragment was amplified with T7 primers (universal sequencing primers for the pET-28a vector) and sequenced. After confirming the correct sequence, positive clones were extracted and transferred into BL21 (DE3) competent cells and induced with 1 mM IPTG (I8070, Solarbio, China) at 16℃ and 160 rpm. The target protein was purified using GE Cytiva columns (17524701, Cytiva, USA) and an AKTA Pure protein purifier (GE Cytiva, Sweden).

## Polyclonal antibody preparation

The Hoc and Soc protein of ΦPNJ-9 were expressed in *E.coli*, and the purified proteins were subcutaneously administered to four-week-old BALB/c mice at a dose of 0.1 mg/mouse. Three immunizations were performed on days 0, 14, and 28. Seven days after the third immunization, blood was collected via eyeball extraction. The blood was allowed to colt at 37℃for 1 h, then rested overnight at 4℃, and centrifuged at 2400 × *g* for 7 min at 4℃. The supernatant was collected to obtain polyclonal antibodies, which were stored at -20℃. Rabbit polyclonal antibodies against the ΦPNJ-9 Hoc protein were prepared using a similar protocol. Phage samples were resolved on 4-20% SDS-PAGE gels (Smart Life Science, China) and transferred to 0.45 μm PVDF membranes. Antibody immunogenicity was assessed by Western blot.

## Hoc antibody blocking assay *in vivo*

Four-week-old BALB/c mice were first treated with 5 g/L streptomycin sulfate in drinking water for 24 h, followed by 24 h fasting period to empty the gastrointestinal contents. Mice were then intragastrically administered 200 μL of 5% sodium bicarbonate to neutralize gastric acid, followed 30 min later by intragastric administration of 200 μL ΦPNJ-9 incubated with Hoc antibody (murine Hoc polyclonal antibody, mixed with phage at a 1:10 ratio and incubated at 37℃ for 1 h) or ΦPNJ-9 incubated with PBS (PBS mixed with phage at a 1:10 ratio and incubated at 37℃ for 1 h) (phage titer: 1.278×10^9^ PFU/mL). After 12 h and 24 h, caecal and colonic tissues were collected, weighed, homogenized using a tissue homogenizer, and centrifuged at 4000 × *g* for 10 min. The supernatants were collected to assess phage potency.

## Affinity test of phage to mucus *in vivo*

Group 1 (NAC+ΦPNJ-9) and group 2 (ΦPNJ-9) were treated as follows. Group 1 mice received 5 mg/mL NAC (HY-B0215, MCE, USA) via intragastric administion at a dose of 200 μL/mouse for 11 consecutive days. On the 9^th^ day, mice were treated with 5 g/L streptomycin sulfate in their drinking water for 24 h, followed by a 24 h fasting period on the 10^th^ day to empty the gastrointestinal contents. On the 11^th^ day, after 2.5 h of NAC gavage, gastric acid was neutralized by gavage with 200 μL of 5% sodium bicarbonate, followed 30 min later by gavage with 200 μL of ΦPNJ-9 phage (1.12×10^9^ PFU/mL). Group 2 mice were treated with PBS instead of NAC, with all other treatments identical to group 1. After 12 h and 18 h post-phage gavage, caecal and colonic tissues were collected, weighed, homogenized, and centrifuged at 4000 × *g* for 10 min. The supernatants were collected to determine phage titers.

## Adhesion of phage to different intestinal cells

LS174T, HT-29 and Caco-2 cells were cultured in six-well plate. LS174T cells were cultured in MEM medium (C11095500BT, Gibco, China), while HT-29 and Caco-2 cells were cultured in DMEM medium (C11995500BT, Gibco, China). All media were supplemented with 10% fetal bovine serum (FBS, F2442, Sigma, Germany）and 100 μg/mL penicillin-streptomycin（P/S, 15140-122, Gibco, China). Once a monolayer was formed, the medium was removed, and 500 μL of serum-free medium was added to each well. subsequently, 500 μL of ΦPNJ-9 (5×10^9^ PFU/mL) was added, and the cells were incubated at 37℃for 2 h. After incubation, the supernatant was discarded, and the cells were washed 3 times with PBS. Finally, 1 mL PBS was added to each well, and the cells were scraped off to determine the phage titer.

## Construction and expression of mutant Hoc protein

Primers for site-directed mutagenesis were designed using the online tool from Nanjing Novozymes Biotechnology Co., Ltd (https://crm.vazyme.com/cetool/singlefragment.html), with all designed primers listed in Table S2. The pET-28a plasmid vector containing the ΦPNJ-9 *hoc* gene was used as a template for PCR amplification of the mutated *hoc* gene. *Dpn* I enzyme (1235S, Takara, Japan) was added to the PCR reaction solution and incubated at 37℃ for 1 h to specifically excise the methylated template DNA strand, retaining the newly synthesized strand. The resulting plasmid was then transformed into DH5α competent cells, and the subsequent steps followed the standard procedure as for prokaryotic protein expression.

## Removal of fucose or sialic acid residues from MUC2

LS174T cells were cultured in 10-cm dishes until a monolayer was formed. The culture medium was discarded, and the cells were washed once with PBS. Next, 500 μL Western and IP cell lysate (P0013, Beyotime, china) was added to the cell, which were gently shaken every 5 min at 4℃ to ensure even distribution. After 20 min, the cells were scraped off, and the lysates were centrifuged at 13500 × *g* for 10 min at 4℃ to abtain LS174T cell lysates. To remove fucose or sialic acid residues from MUC2, α1-2,4,6 fucosidase O (P0749S, NEB, USA) or α2-3,6,8,9 neuraminidase A (P0722S, NEB, USA) was added to the lysates and incubated at 37℃ for 1 h. Protein A/G Magnetic beads (B23202, bimake, USA) and MUC2 antibody (547659, biorbyt, UK) were incubated at room temperature for 30 min, and the α1-2,4,6 fucosidase O or α2-3,6,8,9 neuraminidase A-treated lysates were added. The mixture was incubated overnight at 4℃ to capture MUC2 protein depleted of fucose or sialic acid residues.

## Adhesion of Hoc protein and its mutant to LS174T cells

LS174T cells were cultured in six-well plates until a confluent monolayer was formed. The culture medium was then discarded. To remove fucose residue from MUC2, 4 μL of α1-2,4,6 fucosidase O (P0749S, NEB, USA) was added and incubated at 37℃ for 1 h. Following incubation, 600 μL of serum-free MEM medium was added to each well. Subsequently, 200 μL of wild-type or mutant Hoc protein (1 mg/mL) was applied to the cells, while the negative control group received 200 μL of PBS. After a 1.5 h incubation at 37℃, the supernatant was discarded, and the cells were washed 3 times with PBS. Cell lysis was performed by adding 100 μL of Western and IP cell lysate (P0013, Beyotime，china) to each well. The samples were collected for Westen blot analysis to assess cells adhesion. Primary antibodies used were a Rabbit-derived Hoc polyclonal antibody (1:1000 dilution) and an MUC2 antibody (T56761, abmart, China, 1:1000 dilution). Secondary antibody: Goat Anti-Rabbit IgG HRP antibody (M21002, abmart, China, 1:5000 dilution). Band intensities were quantified using ImageJ software.

## Co-immunoprecipitation assay

To perform co-immunoprecipitation, 5 μL of MUC2 antibody was incubated with 50 μL of Protein A/G Magnetic Beads on a rotary mixer for 30 min at room temperature. For the negative control, 5 μL of IgG antibody was incubated with 50 μL of Protein A/G Magnetic Beads under the same condition. Following antibody incubation, the beads were magnetically separated, aspirated, and discarded using a magnetic stand, then washed three times with pre-chilled PBS. LS174T cell lysate (500 μL) was added to the beads and incubated on a rotary mixer for 7-8 h at 4℃. After incubation, magnetic separation was performed, and the supernatant was discarded. The beads were washed 6 times with pre-chilled PBS. Next, 600 μL of wild-type Hoc protein, mutant Hoc protein, or Hoc domains protein (0.8 mg/mL) was added to the beads pellet and incubated overnight at 4℃on a rotary mixer. The mixture was then centrifuged at 60 × *g* for 30 seconds at 4℃. The precipitate was retained, and the supernatant was discarded by magnetic separation. The beads were washed 14 times with NP-40 lysis buffer (N8032, Solarbio, China) containing 1% PMSF (P0100, Solarbio, china) and 2% Tween-20 (T8220, Solarbio, china). Finally, the bead pellet was resuspended in 40 μL of PBS, and the presence of different Hoc proteins and MUC2 was analyzed by Western blotting.

## M13 phage display

primers for the synthesis of M13 phage modifications were designed using online tools from Nanjing Novozymes Biotechnology Co., Ltd (<https://crm.vazyme.com/>cetool/singlefragment.html), with all designed primers listed in Table S2. The *hoc* domain 2 gene was amplified by PCR using ΦPNJ-9 as a template. The pCANTAB 5E vector was digested with the endonucleases *Sfi* I (R0123V, NEB, china) and *Not* I (R0189V, NEB, china). The *hoc* domain 2 gene was then ligated into the double-digested pCANTAB 5E vector by homologous recombination. The recombinant plasmid was transferred into TG1 chemically competent cells, and positive clones were screened on LB-2% Glu-Amp plates. Positive clones were cultured in 2×YT-2% Glu-Amp medium, and the target fragment was amplified and sent for sequencing.

A positive bacterial clone was inoculated into 200 mL of 2×YT-2% Glu-Amp medium and cultured an OD_600_ of 0.6-0.8. Subsequently, 30-50 μL of helper phage M13K07 was added, and the mixture was incubated at 37℃ for 40 min. The culture was centrifuged at 3000 × *g* for 20 min at 25℃, the supernatant was discarded, and the pellet was resuspended in 400 mL of 2×YT-Kana+Amp medium. The culture was incubated at 225 rpm for 14 h at 37℃. The bacterial culture was then centrifuged at 4500 × *g* for 35 min at 4℃, and the supernatant was collected.

The supernatant was mixed with 80 mL of PEG6000-NaCl solution (40g PEG6000, 29.2 g NaCl, 200 mL H_2_O , autoclaved at 121℃ for 15 min) and incubated on ice for 12-24 h. The mixture was then centrifuged at 6000 × *g* for 1 h at 4℃, and the supernatant was discarded. The pellet was resuspended in PBS and incubated on a shaker at 4℃ for 12-14 h. Finally, the mixture was centrifuged at 13,000 × *g* for 20 min at 4℃, and the supernatant was collected and stored in the dark at 4℃ to obtain the recombinant M13 phage.

## Immunofluorescent staining *in vivo*

To investigate the adhesion of ΦPNJ-9 in the mouse intestine, mice were randomly assigned to one of four groups: (1) control group: intragastrically administered PBS (200 μL/mouse); (2) phage group: intragastrically administered 200 μL of ΦPNJ-9 phage (1×10^9^ PFU/mL); (3) antibody-blocked phage group: intragastrically administered 200 μL of ΦPNJ-9 phage pre-blocked with Hoc antibody (1×10^9^ PFU/mL); and (4) NAC-treated group: mice were first administered NAC (1 mg/mouse) intragastrically for 11 consecutive days, followed by 200 μL of ΦPNJ-9 phage (1×10^9^ PFU/mL). After 7 h of treatment, colonic tissues were harvested and processed for paraffin embedding and fluorescence staining.

To assess the adhesion of M13 phage displaying domain 2 of the ΦPNJ-9 Hoc protein in the intestine, mice were randomly divided into 2 groups and gavaged with 200 μL (1×10^11^ PFU/mL) of either M13-stuffer phage or M13-domain2 phage. 7 h post-gavage, colon tissues were collected, paraffin-embedded, and subjected to fluorescence staining.

## Preparation of paraffin sections and fluorescence staining

Paraffin-embedded sections were deparaffinized and washed sequentially in deparaffinizing solution, absolute ethanol, and distilled water. Slides were then washed three times for 5 min each on a destaining shaker in PBS. After slight shaking, the tissue was circled with a histochemical pen. Sections were blocked with 3% BSA for 30 min. Two primary antibodies, Anti -MUC2 Rabbit pAb (GB11344-100, Servivebio, China) and a mouse-derived Soc polyclonal antibody, were mixed and applied dropwise to the sections. The slides were incubated flat in a humidified chamber overnight at 4℃.

Following incubation, the slides were washed three times for 5 min each in PBS on a destaining shaker and incubated with the corresponding secondary antibody CY3-labeled goat anti-rabbit IgG (GB21303, Servicebio, China) and CY5-labeled goat anti-mouse IgG (GB27301, Servicebio, China) for 50 min at room temperature, protected from light. After washing three times in PBS, the slides were stained with DAPI (G1012, Servivebio, China) for 10 min at room temperature in the dark. Following three PBS washes, the slides were treated with autofluorescence quencher solution B (G1221, Servivebio, China) for 5 min and rinsed in running water for 10 min. Finally, the slides were mounted with anti-fluorescence quenching mounting medium (G1401, Servivebio, China), and images were captured and analyzed using CaseViewer software.

The immunofluorescence procedures for recombinant M13 phage was similarly, with the primary antibody being anti-M13 PVIII antibody (MVV05401, Antibodysystem, France) and the secondary antibody Alexa Fluor 488-conjugated goat anti-mouse IgG (GB25301, Servicebio, China).

## Effect of phage and Hoc protein on MUC2 expression

LS174T cells were cultured in six-well plates until a confluent monolayer was formed. The culture medium was discarded, and 900 μL of serum-free MEM medium was added to each well. Subsequently, 100 μL of Hoc protein (1.5 mg/mL) or phage solution (2.5×10^9^ PFU/mL) was added to the cells, which were incubated at 37℃ for 1, 2, or 3 h. After incubation, total cellular RNA was extracted using the FastPure Cell/Tissue Total RNA Isolation Kit V2 (RC112-01, Vazyme, China). cDNA was sythesized by reverse transcription using HiScript II Q RT SuperMix for qPCR (+ gDNA wiper) (R323-01, Vazyme, China). PCR was performed using AceQ qPCR SYBR Green Master Mix (High ROX Premixed) (Q141-02, Vazyme, China) to quantify the transcript levels of *Muc2* and *gapdh* (internal reference gene). Data analysis was performed using the standard 2^−△△Ct^ method. The primers used are listed in Table S2.

For protein expression analysis of MUC2, LS174T cells were incubated with Hoc protein or phage for 1, 2 or 3 h. Folowing incubation, 100 μL Western and IP cell lysate (P0013, Beyotime，china) was added to each well for protein extraction. Western blotting was performed to detect MUC2 protein expression. The primary antibody used was MUC2 antibody (T56761, abmart, China) at a dilution of 1:1000, and the secondary antibody was Goat Anti-Rabbit IgG HRP (M21002, abmart, China) at a dilution of 1:5000. GAPDH, as an internal control, was detected using the primary antibody (AP0063, Bioworld Technology, China) at a dilution of 1:1000 and the secondary antibody Goat Anti-Rabbit IgG HRP (M21002, abmart, China) at a dilution of 1:5000.

## Prevention experiment in mice

Four-week-old BALB/c mice were randomly assigned to 3 groups: (1) ΦPNJ-9+ETEC; (2) Hoc antibody-blocked ΦPNJ-9+ETEC; and (3) PBS+ETEC. Mice were treated with 5 g/L streptomycin sulfate in drinking water for 24 h, followed by a 24 h fasting period. Gastric acid was neutralized by gavage with 200 μL of 5% sodium bicarbonate, administered 10 h before the end of the fasting period. Thirty munites later, group 1 mice received 200 μL of free ΦPNJ-9 (8×10^10^ PFU/mL), group 2 mice received 200 μL of Hoc antibody-blocked ΦPNJ-9 (8×10^10^ PFU/mL), and group 3 mice received 200 μL of PBS. After the fasting period, 200 μL of 5% sodium bicarbonate was administered again intragastrically. 30 min later, all mice were intragastrically challenged with ampicillin-resistant ETEC SH232 (2×10^7^ CFU/mouse). After 7 h, intestinal mucus and contents were collected. Phage loads in the intestinal mucus and contents were detected using the double-layer agar method, while bacterial loads were assessed on LB-Amp plates.

## One-step growth curve

The optimal multiplicity of infection (MOI) for ΦPNJ-9 and ΦPNJ-6 was determined through experiments. Equal volumes (1 mL each) of phage and host strain were mixed according to the optimal MOI, incubated at 37℃ for 10 min, and then centrifuged at 10,000 × *g* for 10 min. The supernatant was discarded, and the pellet was resuspended with 5 ml of LB liquid medium preheated at 37℃. The suspension was immediately placed in a shaker for incubation. Samples were taken every 10 min for a total of 120 min to determine phage titer, these date were used to construct a one-step growth curve and calculate the phage lysis. The phage lysis rate was calculated as the ratio of the mean bacteriophage plaque number during lysis to the mean bacteriophage plaque number during the latent period.
