# Supplementary figures and images for: Escherichia coli phage ΦPNJ-9 adheres to mucus via a variant Hoc protein
Source: J Virol. 2024 Dec 26;99(2):e01789-24. doi: 10.1128/jvi.01789-24 (PMC11853027; doi:10.1128/jvi.01789-24)

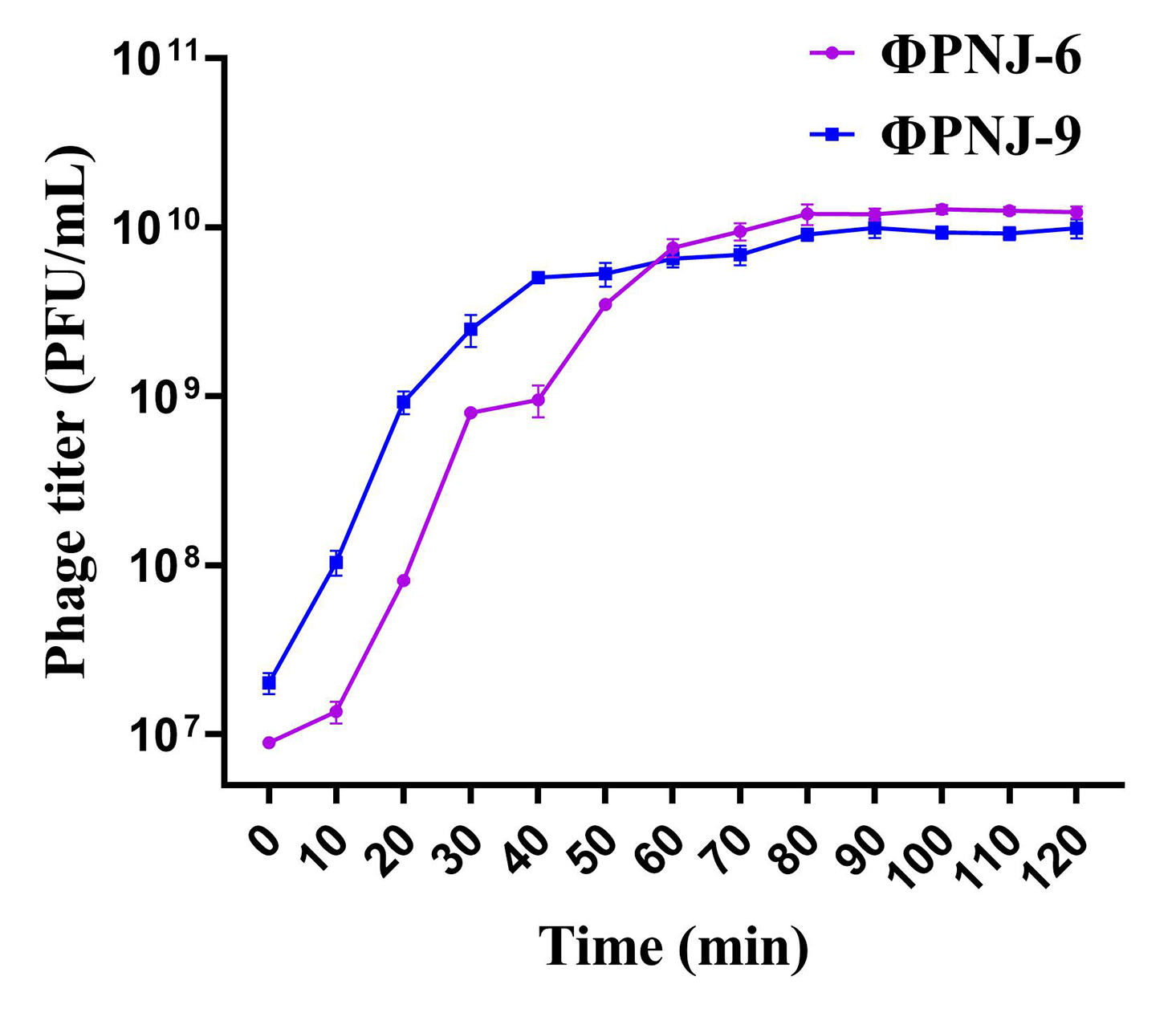

Supplement: Figure S1 — One-step growth curve. [file jvi.01789-24-s0002.tif]
