# Supplementary material for: Escherichia coli phage ΦPNJ-9 adheres to mucus via a variant Hoc protein
Source: J Virol. 2024 Dec 26;99(2):e01789-24. doi: 10.1128/jvi.01789-24 (PMC11853027; doi:10.1128/jvi.01789-24)
Supplement: Supplemental legend — Legend for Fig. S1. [file jvi.01789-24-s0003.docx]

**Figure legends of Fig S1**

**Fig S1 One-step growth curves of phages ΦPNJ-9 and ΦPNJ-6.**

The replication dynamics of ΦPNJ-9 and ΦPNJ-6 were analyzed using ETEC SH232 as the host bacteria. Phage-host mixtures were prepared at the optimal MOI, incubated at 37°C, and subjected to one-step growth experiments. Following adsorption and centrifugation, the pellet was resuspended in prewarmed LB medium and incubated with shaking. Samples were taken every 10 min for 120 min to measure phage titers. Data are presented as means from three independent experiments, with error bars representing standard deviations.
